# High Pressure Processing vs. Thermal Pasteurization of Whole Concord Grape Puree: Effect on Nutritional Value, Quality Parameters and Refrigerated Shelf Life

**DOI:** 10.3390/foods10112608

**Published:** 2021-10-28

**Authors:** Yuanyuan Li, Olga I. Padilla-Zakour

**Affiliations:** Department of Food Science, Cornell University, Stocking Hall, Ithaca, NY 14853, USA; yl645@cornell.edu

**Keywords:** high-pressure processing, Concord grape, microbial inactivation, physicochemical properties, antioxidant activity, nutritional value, sensorial attributes

## Abstract

High-pressure processing (HPP) is utilized for food preservation as it can ensure product safety at low temperatures, meeting consumers’ demand for fresh-like and minimally processed products. The purpose of this study was to determine the effects of HPP (600 MPa, 3 min, 5 °C) and pasteurization by heat treatment (HT, 63 °C, 3 min) on the production of a novel whole Concord grape puree product (with skin and seeds, no waste), and the shelf-life of the puree under refrigerated storage (4 °C). Microbial load, physicochemical properties, phenolic content and antioxidant activity, composition and sensorial attributes of puree samples were evaluated. HPP- and HT-treated purees were microbiologically stable for at least 4 months under refrigeration, with less microbial growth and longer shelf life for HPP samples. HPP and HT samples had similar levels of phenolic contents and antioxidant activities throughout the 4-month refrigerated storage period, even though HPP retained >75% PPO and POD enzyme activities while those of HT were less than 25%. Inclusion of seeds in the puree product significantly increased the fiber, protein, total fatty acid, and linoleic acid contents. Sensory results showed that HPP-treated puree retained more fresh-like grape attributes, had better consistency, and showed significantly higher ratings in consumer overall liking, product ranking, and purchase intent than the HT puree (*p* < 0.05).

## 1. Introduction

Grape is one of the most favored fruits worldwide and has the highest total value of production. Consumer demand for both table grapes and processed grape products has driven the total global grape production to 79 million tons in 2018, an increase of 4 million tons compared to 2014 [1]. In addition to its palatable characteristics, the health-promoting functions of grape are another aspect highly valued by the consumers. Researchers have assessed the health benefits of grape and grape products in both animal and human studies [2]. Flavonoids are the most abundant phytonutrients found in grapes. Anthocyanins are a subclass of flavonoids which are responsible for the attractive skin color in red grape varieties [3].

The Concord grape is a dark purple to blue colored variety belonging to *Vitis labrusca*. The health-promoting properties of Concord grape have been reported as cardiovascular protection [4,5,6,7,8], neuroprotection [9,10], and antiaging [11,12] and antitumoral effects [13]. Compared to culinary grape products, grape-derived commercial products, such as grape seed extracts, grape seed oil, and grape powder (grape skin dietary fiber), are rapidly expanding in the marketplace as they can be classified as high-value-added nutraceuticals or cosmetics. This is attributed to the highly valued phenolic compounds that can reduce the oxidative stress which leads to many chronic diseases and the deterioration of normal body function. The phenolic compounds are mainly located in the skin (55%) and seeds (44%) of grapes [14], which are considered processing waste or byproducts of the wine- and juice-producing industries. Grape seed extracts have been studied and reported as antimicrobial [15], antitumor [16,17], anticancer [18,19], and anti-inflammation agents to lessen Alzheimer’s disease [20,21]. As the most widely accepted grape product, grape juice has been reported to have the disadvantages of high sugar content and antimicrobial addition for preservation. Wine has been favored for its cardioprotective effect for many years, even though its production can only extract a maximum of 50% of the total phenolic compounds from grape berries. However, there are rising concerns about wine, such as alcohol addiction and the adverse health effects [22]. Alternatively, grape puree can be directly consumed or applied as an ingredient. One aim of this research was to develop a whole Concord grape puree product (including seeds and skin, eliminating waste), to meet consumers’ demand for healthy fruit products. Conventional thermal processing is often applied to preserve food products, especially for highly perishable fruits with short harvest seasons, such as Concord grape. Despite the effective reduction of microbial loads and inactivation of deleterious enzymatic activity, thermal processing can cause adverse effects on fruit product quality. Quality parameters such as color, nutrient content, and sensory attributes are crucial factors that affect consumers’ acceptance, and can be deteriorated during thermal processing [23,24]. Alternatively, nonthermal technologies can meet the consumers’ demand for minimally processed products with clean labeling, high nutrition, and fresh-like appearance and taste. High-pressure processing (HPP) is one of the most widely investigated nonthermal technologies and has been successfully utilized on many food products. HPP ensures food safety by killing vegetative cells of microorganisms via cell membrane rupture under extreme pressure, leading to loss of normal function and integrity [25,26]. Regarding food quality, high pressure affects the morphology and function of macromolecular food components such as proteins, enzymes, lipids and polysaccharides, while low-molecular-weight food components such as vitamins and flavoring and coloring compounds, which are often the important components determining the nutritional and sensorial attributes, are less affected as covalent bonds are not disrupted [27,28].

HPP has been recognized as the most successfully commercialized nonthermal technology in the food industry, and its application on fruit products is rising [29]. Previous research has laid the groundwork for the application of HPP in industry-scale food processing. For instance, raspberry puree showed the smallest anthocyanin degradation rate when pressurized at 200 to 800 MPa during storage at 4 °C [30], and the red color loss was closely associated with the residual enzyme activity after HPP [31]. HPP treatment at 400 to 600 MPa for 5 min provided a microbially safe aronia berry puree product that retained physicochemical properties and nutritional value [32,33]. Similar studies have also been conducted on HPP-treated acidified apple [34], strawberry [35,36,37], blackberry [38], plum [39], pineapple [40], and banana purees [41,42]. Application of HPP on grape products has been mainly reported in studies of grape juice, pomace, and wine [43,44,45,46], whilst no report on grape puree products has been published.

The objective of this study was to assess the effects of HPP and mild heat pasteurization on the quality of whole Concord grape puree during its refrigerated shelf-life. The relevance of this study to the food industry is that it supports sustainable approaches to eliminating waste while providing evidence of the use of HPP to develop a fresh-like and nutritious whole Concord grape puree with extended refrigerated shelf life.

## 2. Materials and Methods

### 2.1. Materials and Chemicals

Fresh Concord grapes (*Vitis Labrusca* L.) provided by Welch’s Foods Inc. (Concord, MA, USA) were harvested in October 2019 from the Finger Lakes region, NY. Grapes were stored in a refrigerated room at 4 ± 1 °C and processed into puree within one week after harvest. The grapes used in this study were from the same batch.

All chemicals used were of analytical grade. Anhydrous gallic acid was obtained from Chem-Impex, Wood Dale, IL, USA. Folin–Ciocalteu’s phenol reagent, ABTS [(2,2′-Azinobis-(3-ethylbenzothiazoline-6-sulfonic acid) diammonium salt)], and methanol were purchased from Sigma-Aldrich, St. Louis, MO, USA. DPPH (1,1-diphenyl-2-picrylhydrazyl radical), Trolox (6-hydroxy-2,5,7,8-tetramethyl-chroman-2-carboxylic acid), and catechol were purchased from TCI America, Portland, OR, USA. Potassium persulfate was purchased from Honeywell Fluka, North Carolina. Triton-100 was purchased from Electron Microscopy Sciences, Hatfield, PA, USA. Poly(vinylpolypyrrolidone) (PVPP), p-phenylenediamine, hydrogen peroxide, sodium chloride, and monobasic and dibasic sodium phosphate were purchased from VWR International, Radnor, PA, USA.

### 2.2. Concord Grape Puree Preparation

**Figure 1:**
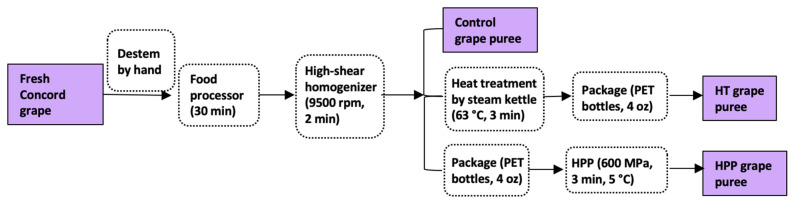
Flow diagram for the production of whole Concord grape puree.

Figure 1 shows the processing procedure followed. Concord grapes were destemmed by hand and then ground using a food processor (R302V, Robot Coupe, Ridgeland, MS, USA) for 30 min. Grape seeds in the puree were broken into small pieces after grinding. Then the slurry was sheared in a Ross high-shear homogenizer equipped with a fine screen stator (HSM-100LSK, Charles Ross & Son, Hauppauge, NY, USA) at 9500 rpm for 2 min to obtain a smooth puree. An ice-water bath was used to maintain the temperature of the grape slurry below 40 °C during processing.

### 2.3. Puree Preservation

#### 2.3.1. High-Pressure Processing (HPP)

For quality and shelf-life studies, samples were processed at the Cornell HPP Validation Center (Geneva, NY, USA), following biosafety level 2 guidelines, which prohibits testing for sensory analysis. HPP-compatible PET bottles (4 oz, Merrimack Valley Plastics, Methuen, MA, USA) filled with sheared puree samples were packed into PET bags and vacuum sealed. Each bag was then bagged and sealed again to prevent any leakage in the HPP system. Packages were loaded into a 55 L commercial high-pressure processing unit (Hiperbaric 55, Hiperbaric, Burgos, Spain) and cold water was used to transmit pressure. Puree samples were pressurized using current industry standards of 600 MPa for 3 min at 5 °C. These HPP parameters (600 MPa, 3 min) are commonly used in the food industry, and have been tested to achieve a greater than 5-log reduction of relevant pathogens in acid/acidified juices/beverages (pH < 4.5) [47]. After HPP, all bags were discarded and bottles were wiped dry. HPP-treated samples for the sensory study were prepared the same way except that the HPP treatment was completed at a commercial food plant, LiDestri Food and Beverage (Rochester, NY, USA) with an industrial HPP unit (Hiperbaric 525, Hiperbaric, Doral, FL, USA).

#### 2.3.2. Heat Treatment (HT)

A mild heat treatment (63 °C, 3 min) was applied for thermal processing to retain fresher attributes. This time and temperature combination was calculated based on a D_52_ value of 23 min and a z-value of 4.8 °C, with an additional 5-fold safety factor to achieve a >5-log reduction process for *E. coli O157:H7, Salmonella,* and *Listeria monocytogenes* [48]. Puree samples were pasteurized for safety in a steam kettle (TDA-10 QT, IL) to achieve >5-log reduction of pertinent pathogens (FDA, 2004), and then immediately cooled in an ice-water bucket. When temperatures dropped below 38 °C, samples were poured into clean 4 oz PET bottles and immediately closed tightly using screw caps.

### 2.4. Refrigerated Storage

Samples used for the sensory study were kept under refrigeration at 4 °C for 1 week, to simulate commercial distribution time required to reach stores, before sensory analysis. Control (untreated puree), HPP-, and HT-treated samples for physicochemical and other quality analyses were stored at 4 ± 1 °C and sampled at 1-month intervals for the shelf-life study for up to 5 months. All samples were prepared according to the flow diagram (Figure 1), except for samples used for the proximate composition analysis.

### 2.5. Microbial Analyses

#### 2.5.1. Total Aerobic Plate Count

Total aerobic plate counts (APCs) were determined by taking puree samples monthly during refrigerated storage. Twenty-five grams of Concord grape puree sample was diluted (1:10 *w*/*w*) in 0.1% sterile peptone water and homogenized using a stomacher (Stomacher 400 Circulator, Seward Medical, London, UK) at 200 rpm for 1 min at ambient temperature. The homogenized solution was then serially diluted in 9 mL of peptone water and pour-plated for APC using Plate Count Agar (PCA, CM0325, Oxoid Limited, Thermo Fisher Scientific Inc., Hampshire, UK), followed by incubation at 30 °C for 48 to 72 h. APC was expressed as log of colony-forming units per gram of puree by fresh weight (log CFU/g FW).

#### 2.5.2. Yeast and Mold Count

Yeast and mold (Y&M) counts were assessed at the same sample interval and using the same preparation procedures as APC analyses, except that Potato Dextrose Agar (PDA, Alpha Biosciences Inc., Baltimore, MD, USA) was used as growth medium. Tartaric acid was added to the PDA medium to adjust the pH to 3.5. Y&M counts were expressed as log CFU/g FW.

### 2.6. Physicochemical Properties Analyses

#### 2.6.1. Total Soluble Solids Content (TSSC)

Total soluble solids content was determined using a portable digital refractometer (model 300055, Sper Scientific, Scottsdale, AZ, USA). Approximately 5 g of grape puree was filtered through Whatman No.4 filter paper and 2 to 3 drops of filtrate were added onto the prism at room temperature to obtain readings, expressed as °Brix.

#### 2.6.2. pH

pH was measured at room temperature using a pH meter (Orion^TM^ 3-star, benchtop pH meter, Thermo Scientific^TM^, Fisher Scientific, Waltham, MA, USA), which was calibrated prior to each measurement with standard phosphate buffers at pH 4 and 7.

#### 2.6.3. Titratable Acidity (TA)

TA was determined according to Iland [49] (pp. 39–43) with some modifications. After calibrating the pH meter using the pH 4 and pH 7 buffers, a diluted solution containing 5 g of grape puree sample and 45 mL of distilled water was titrated with 0.1 mol/L sodium hydroxide to pH 8.2 using an autotitrator (Mettler compact G20, Mettler-Toledo, LLC, Columbus, OH, USA). Results were calculated based on Equation (1) and expressed as % tartaric acid (% gram tartaric acid equivalence per gram of grape puree):

(1) $$\mathrm{Tartaric}\;\mathrm{acid}\;(\%,\;w/w)=\frac{V_{NaOH}(L)\times 0.1(\frac{\mathrm{mol}}{L})\times 75(\frac{g}{\mathrm{mol}})}{m_{puree}(g)}\times 100$$

#### 2.6.4. Color Measurement

Color components {L* (lightness), a*(greenness [−] to redness [+], and b* (blueness [−] to yellowness [+])} of puree samples were determined using a Hunter colorimeter (Labscan XE, Hunter Associates Laboratory, Inc., Reston, VA, USA) in reflection mode. The colorimeter was first standardized with a white tile and samples were measured in a 10 mm path-length quartz cuvette. Two measurements for each sample were conducted, and an average value was reported as the color result of the sample. The values of the absolute color difference of a sample were calculated according to Equation (2) as shown below:

(2) $$∆E=\sqrt{{\left(L-L_{0}\right)}^{2}+{\left(a-a_{0}\right)}^{2}+{\left(b-b_{0}\right)}^{2}}$$

where $L_{0}$, $a_{0}$, and $b_{0}$ are the color measurements of fresh-made control puree samples; *L*, *a*, and *b* are the color measurements of HPP- or HT-treated puree samples.

The browning index (*BI*) was analyzed according to Palou et al. [41] and calculated using Equation (3):

(3) $$BI=\frac{100\times (x-0.31)}{0.172}$$

where $x=\frac{a+1.75\times L}{5.645\times L+a-3.012\times b}$ in Equation (3).

#### 2.6.5. Particle Size Distribution (PSD), Serum Separation, and Viscosity

The particle size distribution was measured using a laser diffraction particle size analyzer (Malvern Mastersizer 2000, Malvern Instruments Ltd., Westborough, MA, USA). Distilled water was used as a dispersant and the samples were measured when the concentration of added sample reached 5% obscuration. The particle size distribution profiles were measured in triplicate.

Serum separation rate (SSR) was measured as an indicator of syneresis via the centrifugation method described by Eliasson and Kim [50]. Ten grams of puree sample was loaded into a 50 mL centrifuge tube and centrifuged at 6000 rpm for 10 min at room temperature (Centrifuge 5810R Eppendorf, CT). Supernatant was decanted and the weight of remaining solids was recorded. SSR was calculated based on Equation (4):

(4) $$\mathrm{SSR}\;(\%,\;w/w)=\frac{\left(m_{t}-m_{r}\right)}{m_{t}}\times 100$$

where *m_t_* is the total puree weight before centrifugation and *m_r_* is the remaining solids weight after centrifugation.

Viscosity was measured using a Brookfield DV-III Ultra programmable rheometer (Brookfield Engineering Laboratories, Ltd., Middleborough, MA, USA) with a V-73 spindle. Samples were equilibrated to room temperature before measuring at 250 rpm for 3 min.

### 2.7. Phenolic Content and In Vitro Antioxidant Activity

#### 2.7.1. Extraction of Total Phenols and Anthocyanins from Whole Concord Grape Puree

The extraction procedure was based on the methods reported by Iland [49] (pp. 45–48) and Jensen et al. [51] with some modifications. Generally, fresh whole Concord grape puree was mixed with acidified methanol (1% HCl, *v*/*v*) at a 1:1 ratio (*w*/*v*). After vortexing the mixture for 1 min, tubes were put into a 40 °C water bath for 30 min. The supernatant was transferred into new vials after centrifugation (12,000× *g*, 5 min). The supernatant was diluted 5- to 10-fold using distilled water, and then used as total phenols and anthocyanins solution for future determination. Puree weight ($m_{p}$) and supernatant volume ($V_{s}$) were recorded for calculation.

#### 2.7.2. Total Phenolic Content (TP)

Total phenolic content was determined via Folin–Ciocalteau colorimetric assay according to the method reported by Waterhouse [52] with minor modifications. Generally, 20 μL of diluted extract was mixed with 1580 μL DI water and 100 μL Folin–Ciocalteau reagent. The mixture was vortexed and incubated at room temperature for 6 min. After incubation, 300 μL of 20% (*w*/*v*) sodium carbonate solution was added and gently vortexed before incubating at room temperature for 2 h in the dark. Absorbance was measured at 765 nm using a Genesys UV-visible Spectrophotometer (10S, Thermo Fisher Scientific, Waltham, MA, USA). Gallic acid solutions (0 to 500 mg/L) were used to determine the standard curve. Results were expressed as gallic acid equivalent (GAE) mg/g of fresh weight of whole Concord grape puree. Calculation was carried out according to Equation (5):

(5) $$\mathrm{GAE}(\frac{\mathrm{mg}}{g})=\frac{C(\frac{\mathrm{mg}}{L})\times V_{s}(L)}{m_{p}(g)}$$

#### 2.7.3. Total Monomeric Anthocyanin Content (TMA)

Total monomeric anthocyanin content was determined using the method described by Lee et al. [53]. Briefly, 200 μL extracts were separately diluted 10-fold with pH = 1.0 (0.025 M, potassium chloride) and pH = 4.5 (0.4 M, sodium acetate) buffers. The mixture was gently vortexed and equilibrated at room temperature for 20 min. The blank was prepared using DI water. Absorbance readings were taken at both 520 nm and 700 nm using the Genesys UV-visible Spectrophotometer. Results were calculated using Equation (6) and expressed as cyanidin-3-glucoside equivalent (CGE):

(6) $$\mathrm{CGE}\;(\mathrm{mg}/\mathrm{kg})=\frac{A\times M_{w}\times DF\times 10^{-3}\times V_{s}}{\varepsilon \times L\times m_{p}}$$

where $A=(A_{520\mathrm{nm}}-A_{700\mathrm{nm}})pH_{1.0}-(A_{520\mathrm{nm}}-A_{700\mathrm{nm}})pH_{4.5}$; *M_W_* (molecular weight) of cyd-3-glu = 449.2 g/mol; *DF* (dilution factor) = 10; *ε* is the molar extinction coefficient = 26,900 $L^{-1}\times {\mathrm{cm}}^{-1}\times {\mathrm{mol}}^{-1}$ for cyd-3-glu; *L* (pathlength) = 1 cm; $V_{s}$ is the puree extraction volume (mL) and $10^{-3}$ is the conversion of mL to *L*; and $m_{p}$ is the fresh puree weight (g) used for extraction.

Results were calculated and expressed as mg CGE/kg of fresh weight of whole Concord grape puree (mg/kg as CGE).

#### 2.7.4. In Vitro Total Antioxidant Activity

The in vitro total antioxidant activity was determined using DPPH and ABTS assays according to the description of Brand-Williams et al. [54] and Re et al. [55] with some modifications. Briefly, DPPH radical solution was prepared at a concentration of 0.2 mM in methanol, and the absorbance of DPPH solution was adjusted to 0.900 ± 0.050 at 517 nm before testing. ABTS solution was prepared in methanol at the concentration of 7 mM. Potassium persulfate powder was then added to the ABTS solution at a final concentration of 2.45 mM in the mixture. The mixture was left at room temperature for 12–16 h to generate stable radicals. The absorbance of ABTS^•^ solution was adjusted to 0.700 ± 0.050 at 734 nm before using. The puree extracts as described in Section 2.7.1 were used for the DPPH and ABTS assays. The supernatant of puree extract (100 μL) was mixed with 900 μL DPPH^•^ solution and measured at 517 nm after 30 min equilibration under dark and room temperature conditions. For the ABTS assay, 50 μL diluted supernatant (dilution factor = 10) was mixed with 2 mL ABTS^•^ solutions and determined spectrophotometrically after 6 min sitting in a dark environment at room temperature. The blank was prepared in the same way, except that DI water was used instead of puree supernatant. Trolox standards (ranging from 0 to 800 μM) were prepared by diluting the stock solution (2.5 mM). Radical scavenging capacity was expressed as TEAC (Trolox equivalent antioxidant capacity) in µmol/g of FW (fresh weight) puree sample.

#### 2.7.5. Enzymatic Activities: Polyphenoloxidases (PPO) and Peroxidases (POD)

Enzyme extraction and assays were carried out to determine the activities of PPO and POD according to Garcia-Palazon et al. [31] with some modifications. Enzyme extraction solution was prepared by mixing 4% (*w*/*v*) PVPP, 1% (*v*/*v*) Triton X-100, and 1 M NaCl with 0.2 mol/L sodium phosphate buffer (pH 6.5). The extraction solution (4.5 mL) and 4.5 g of grape puree sample were mixed vigorously and homogenized for 3 min. The mixture was then centrifuged at 10,000 rpm for 30 min at 4 °C. The supernatant was collected and used as crude enzyme extract in the PPO and POD assays.

For the PPO assay, catechol solution (3 mL, 0.07 M) was prepared by adding catechol powder to 0.05 M sodium phosphate buffer (pH 6.5), and 500 μL of enzyme extract was added to start the reaction. The absorbance at 420 nm was monitored for 3 min and readings were recorded every 30 s. The blank was prepared in the same way except that DI water was used instead of enzyme extract.

For the POD assay, 200 μL of enzyme extract was mixed with 1.5 mL 0.05 M sodium phosphate buffer (pH 6.5) and 200 μL of 10 g/L p-phenylenediamine in 0.05 M sodium phosphate buffer (pH 6.5). To start the reaction, 200 μL 1.5% (*w*/*v*) hydrogen peroxide was added. Readings were recorded at 485 nm using a spectrophotometer. The initial liner region of the absorbance–time curve was used for the enzyme activity analyses.

Both PPO and POD results were shown as the residual activity (RA) in % according to Equation (7):

(7) $$\mathrm{RA}=\frac{A_{t}}{A_{0}}\times 100\%$$

where *A_t_* is the enzyme activity of treated samples and *A*_0_ is the enzyme activity of control samples.

### 2.8. Proximate Composition Analysis

Whole Concord grape purees made with seeds (control, HPP, and HT samples as described in Section 2.3) were used for proximate composition analysis. Additionally, a control sample without seeds was prepared using 1.5 kg Concord grapes that were manually deseeded and then processed into puree. Four groups of samples, namely untreated control with seeds (C/W), untreated without seeds (C/O), HPP-treated with seeds (HPP/W), and HT-treated with seeds (HT/W), were prepared in triplicate for composition analysis. Moisture and dry matter of the fresh puree samples were determined via the oven drying method (930.15, AOAC). Total ash was determined via 942.05, AOAC method. Water-soluble carbohydrates (WSC) were determined spectrophotometrically after acid hydrolysis and colorimetric reaction with potassium ferricyanide [56]. Mineral contents were determined using a Thermo iCAP 6300 inductively coupled plasma radial spectrometer after sample digestion using a CEM microwave accelerated reaction system (MARS6). Crude fiber and crude protein were analyzed according to AOAC 954.02 and AOAC 992.23, respectively [57]. Total fatty acid profiles were analyzed according to O’Fallon et al. [58]. Fatty acid methyl ester (FAME) synthesis was conducted in the presence of up to 33% water. Samples were permeabilized and hydrolyzed for 1.5 h at 55 °C in 1 N KOH in MeOH containing C13:0 as an internal standard. After neutralization of KOH, samples were methylated by H_2_SO_4_ catalysis for 1.5 h at 55 °C. Hexane was then added to the reaction tubes, which were vortex-mixed and centrifuged. The hexane layers were pipetted into gas chromatography vials and then analyzed using a thermo trace 1310 gas chromatograph fitted with a Supelco SP-2560, 100 m × 0.25 mm × 0.20 µm capillary column and a flame ionization detector. All composition analyses were performed at Dairy One Co-Op, Inc., Ithaca, NY, USA.

### 2.9. Sensory Study of HPP- and HT-Treated Purees

A total of 101 untrained panelists were recruited to participate in the sensory evaluation of HPP- and HT-treated whole Concord grape purees. After processing, the puree samples were kept at 4 °C for 1 week before being transported to the Cornell Sensory Evaluation Center (Ithaca, NY, USA). Samples were stored refrigerated before being poured into 2 oz plastic cups coded blindly with three random digits and covered with lids. Panelists were asked to assess the consumer liking of appearance, aroma, texture, flavor, and overall liking using a 9-point hedonic scale rating. Intensity of sweetness, sourness, bitterness, smoothness, flavor, authenticity of Concord grape, and purchase intent were assessed based on 5-point scales. Intensity of color was evaluated using a 9-point red color board with different shades (1 = darkest). A “just about right” (JAR) question was added to assess color preference (1 = too dark, 2 = moderate dark, 3 = just about right, 4 = moderate light, 5 = too light). The evaluation concluded with a preference ranking test between the HPP- and HT-treated samples. This study was conducted under the approval and requirements of the Institutional Review Board of Cornell University.

All samples were served based on a randomized design and following all hygienic and procedural guidelines provided by the Sensory Evaluation Center. Water and crackers were provided for all panelists to consume between samples to reduce the influence from previous samples. Data were collected and analyzed using the REDJADE^®^ Sensory Software (RedJade Software Solutions, LLC, Redwood, CA, USA).

### 2.10. Statistical Analysis

All results are presented as mean values of the data from experiments performed in triplicate. Data are presented as mean ± standard deviation (SD). Data were analyzed using Student’s *t*-test or one-way ANOVA as appropriate. Significant differences among mean values were determined by the Tukey’s post hoc test following the one-way ANOVA test using SPSS (SPSS statistics, version 22.0, IBM, Armonk, NY, USA). Correlation coefficients were determined by Pearson’s correlation test with SPSS.

## 3. Results and Discussion

### 3.1. Microbial Counts in Whole Concord Grape Puree during 5 Months Refrigerated Storage

**Figure 2:**
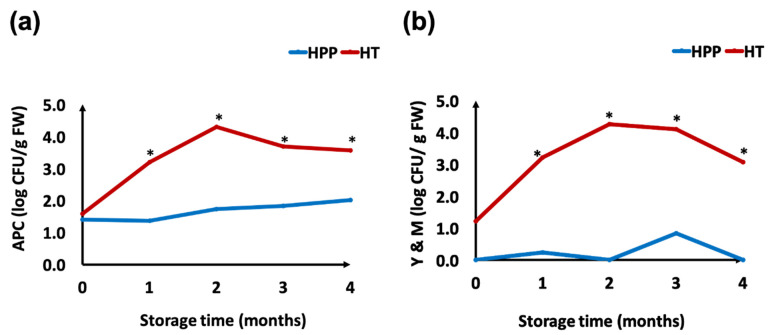
Microbial counts of high pressure processing (HPP, 600 MPa, 3 min) and heat treated (HT, 63 °C, 3 min) puree samples during a 4-month refrigerated storage period: (**a**) total aerobic plate counts (APC); (**b**) yeast and mold counts (Y&M). Denotation of “*” indicates significant differences between treatments at each sampling point.

According to FDA Juice HACCP regulations (FDA, 2004), fruit juice and its related products should achieve a >5-log reduction of potential pathogens after pasteurization to ensure microbial safety. Petrus et al. [59] reported that moderate HPP treatment (400 MPa, 2 min) was sufficient to achieve the 5-log reduction in the pathogen challenge test (mixed cocktails of *Escherichia coli* O157:H7, *Salmonella enterica*, and *Listeria monocytogenes*) in Concord grape juice; thus, the applied 600 MPa for 3 min industrial process met the safety regulatory requirements. The effect of HPP on microbial counts of puree samples during storage at 4 °C is shown in Figure 2. The mean values of APC and Y&M counts in the fresh-made untreated puree were 6.33 and 6.31 log CFU/g FW respectively. Both HPP (600 MPa, 3 min at 5 °C) and HT (63° C, 3 min) treatments were able to cause a 5-log reduction in the fresh-made samples. HPP treatment was able to reduce the Y&M count to a level below the minimum detection limit, which was less than 1.0 log CFU/g FW, while HT treatment decreased the Y&M count to 1.22 log CFU/g FW. The results were consistent with Chang’s findings [44], which reported that HPP- (600 MPa, 3 min) and heat-treated (90 °C, 1 min) white grape juices had similar values in APC (1.2 log CFU/mL), coliform, and Y&M counts (<1.0 log CFU/mL) on day 1 and after 20 days storage at 4 °C, indicating that HPP is very effective in eliminating fungi and vegetative cells of bacteria in grape puree, with limited effect on bacterial spores.

During the 5-month refrigerated storage period, the microbial counts in both treatment groups increased gradually, with values in HT samples showing larger increases than that in HPP samples. After 4 months of storage, the APC count in the HPP samples was 2.0 log CFU/g FW and the Y&M count was still under the detectable limit, while the APC and Y&M counts in the HT samples were 3.6 and 3.1 log CFU/g FW, respectively. After 5 months of refrigerated storage, the APC and Y&M counts in the HPP samples were 2.7 and 2.4 log CFU/g FW respectively, while the HT samples showed indications of spoilage (>6 log CFU/g FW) with visible fungal colonies on the surface. The thermal treatment applied in this study was not as effective as HPP in killing spoilage microorganisms, which led to higher increases of microbial count during storage in HT samples. These findings suggest that HPP is a feasible preservation alternative for fruit puree products, being as effective as mild heat pasteurization in reducing microbial populations and extending refrigerated product shelf life. Because HPP is applied to the packaged product, postprocess contamination is eliminated, contributing to the extended shelf life of refrigerated foods.

### 3.2. Physicochemical Properties of Whole Concord Grape Puree

#### 3.2.1. Total Soluble Solids Content (TSSC), pH, and Titratable Acidity (TA) of Whole Concord Grape Puree during 4 Months Refrigerated Storage

The control puree had a water activity of 0.98, pH of 3.35 ± 0.01, TSSC of 18.0 ± 0.1 °Brix, and TA of 1.2 ± 0.1% tartaric acid on day 1. Heat treatment did not cause significant changes to these parameters immediately after processing (pH = 3.33 ± 0.05, TSSC = 18.1 ± 0.2 °Brix, and TA = 1.3 ± 0.1% tartaric acid) and these values remained constant during storage, except for pH. The pH values in HT samples increased to 3.42 ± 0.05 after 4 months of refrigerated storage, due to the observed microorganism growth within the puree. HPP-treated samples had similar values to those in HT samples on day 1 (pH = 3.35 ± 0.01, TSSC = 18.3 ± 0.3 °Brix and TA = 1.1 ± 0.1% tartaric acid) and during storage (data not shown).

#### 3.2.2. Effect of HPP and HT Treatments on Color Changes of Whole Concord Grape Puree

Color and visual appearance, flavor, texture, and nutritional value are four important attributes that indicate the quality of fruit products. Among them, color and visual appearance are the first quality attributes that impact the perception and acceptance by consumers [60]. Anthocyanins in the skin cells are responsible for the unique red, purple, or dark blue colors observed in Concord and other red grape varieties. As anthocyanins are water soluble and sensitive to both heat and pH, the color degradation in Concord grape products during storage could be impacted by mechanical harvesting, postharvest conditions, heating, enzyme addition, enzymatic browning, tartration, and chemical changes [61,62].

The visual appearance of the freshly produced samples can be seen in Supplementary Figure S2. Instrumental color changes of Concord grape puree with or without treatments during refrigerated storage for 4 months are shown in Supplementary Table S1. There were no significant changes in L*, a*, and ∆*E* values in either the HPP- or HT-treated samples on day 1 and the color parameters remained unchanged after refrigerated storage for 4 months. The b* (yellow/blue) value in HT-treated samples, however, was significantly higher compared to the HPP-treated samples, indicating that more yellow to brown colors were generated in the HT-treated puree samples. Mild heat treatment increased the enzyme (mainly PPO)-mediated browning reaction rate during processing, while the oxidation reaction rate was very slow during HPP processing (5 °C) and refrigerated storage (4 °C). Brown pigments can also be generated through nonenzymatic activity, such as Maillard reactions during juice heating [63,64]. As a consequence of the increased b* value, the Browning Index (BI) in HT-treated samples was significantly higher than that in the HPP-treated and control samples on day 1 and during storage. On day 1, ∆*E* changed to a noticeable level (∆*E* > 2) in HT-treated samples, while the change in HPP-treated samples was not noticeable (∆*E* < 1.5) according to the color change threshold reported in previous studies [65,66]. This finding was in accordance with the results reported for strawberry and blackberry purees, which concluded that color changes in pressurized (400–600 MPa, 10–30 °C, 15 min) purees were minor compared to those in thermally treated (70 °C, 2 min) samples [38]. Marszałek et al. [37] reported that thermal processing at 90 °C for 15 min was able to inactivate 97.7% of enzyme activity in strawberry puree, while the residual PPO enzyme activity of HPP-treated (500 MPa, 0 °C, 5 min) strawberry was still as high as 90%. Based on previous puree studies, HPP-treated puree products had higher residual enzyme activity than thermally treated puree, thus HPP-treated puree may only have a transitory shelf-life and more color loss during storage. In our study, the color change (∆*E*) of both HPP- and HT-treated Concord grape purees remained consistent after a refrigerated storage period of 4 months, suggesting that the unique grape puree matrix containing grape skin and seeds was able to preserve the fresh-like color despite of the possible deleterious enzyme activity in HPP-treated Concord grape puree.

#### 3.2.3. Effect of HPP and HT on Particle Size Distribution (PSD), Serum Separation Rate (SSR), and Viscosity of Concord Grape Puree

Grape puree can be utilized as an intermediate ingredient for product development, such as in beverages, jam, jelly, bakery products, yogurt, and ice cream. Understanding the rheological properties, which determine the textural appearance and viscoelastic parameters, is essential in developing new products as they affect the processing conditions (pumping, mixing, evaporation, and pasteurization) and final consumer acceptance (appearance, consistency, and stability) [67]. Parameters such as TSSC, SSR, PSD, and viscosity are good indicators of rheological behaviors in puree products [68,69,70,71,72]. The SSR in HPP-treated puree was 44.3 ± 3.5%, which was similar to the value of the untreated sample but significantly lower than that of the HT-treated samples (Supplementary Figure S1). After 4 months of refrigerated storage, the SSR in HPP-treated puree increased significantly to 65.8 ± 0.3%, while the HT-treated samples gelled, likely due to enzyme activity, impeding the serum separation from the pulp based on the applied centrifugal force. After 4 months of storage, HPP-treated puree had a good consistency which resembled the freshly made untreated puree with good runniness, while the HT puree had large lumps and small amounts of serum syneresis on the surface (Figure S2b). The HPP puree was smooth, lump free, homogenous, and more like a liquidized, thin puree, while the HT puree surface was rough and lumpy, resembling an uneven puree with gelled components, unsuitable for pumping. The visual lumps and syneresis not only affect the appearance, but can also negatively affect processing operations such as pumping, mixing, and filling. Syneresis in the 4-month HT samples indicated that repelling of water and aggregation of grape components were happening, possibly caused by the entanglement of pectin chains after HT treatment, which greatly deteriorated the product’s physical stability [73].

**Figure 3:**
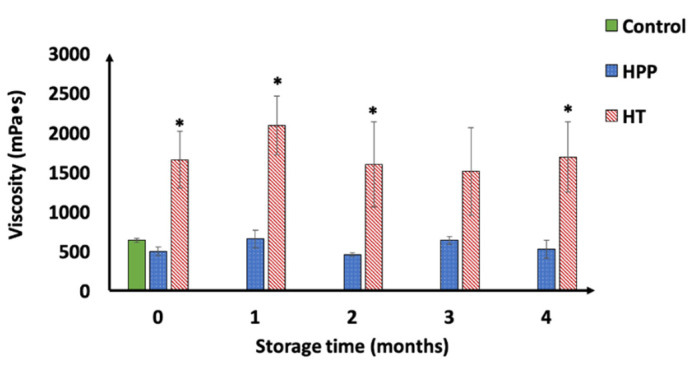
Viscosity changes of control, HPP- (600 MPa, 3 min), and HT-treated (63 °C, 3 min) puree during 4 months of storage at 4 °C. Denotation of “*” indicates significant difference among treatments at each sampling point (*p* < 0.05).

Freshly made grape puree showed a bimodal particle size distribution (Supplementary Figure S2a), regardless of the processing applied, as the puree comprised of a mixture of particles from skins, pulp, and seeds. In HPP-treated puree, a larger proportion of small particles was present, likely due to the physical disruption of the grape components and breakage of cell clusters by the high pressure applied [74]. HT-treated puree had more large particles as a result of aggregation of cell components. After 4 months of storage, the D90 value in HT samples was 294 ± 36 µm, which was significantly higher than that in the HPP samples at 218 ± 35 µm (data not shown). The heat treatment significantly increased the viscosity of the whole Concord grape puree compared to the control and HPP, and the same tendency was also found during storage (Figure 3). The viscosity increase in the HT sample was probably associated with the presence of more large particles and the structural changes of polysaccharides and pectic substances which, respectively, constitute 30% and 20% of the grape pomace. On one hand, apparent viscosity increased as particle size increased, an effect also seen in tart cherry puree and apple puree [75,76]. On the other hand, polysaccharides were hydrated and swollen during heat treatment, which could lead to viscosity increase and polysaccharide aggregation. A low temperature of heat treatment (50 °C to 80 °C) activates the endogenous pectin methylesterase (PME) which results in pectin demethoxylation and cross-linking with calcium ions. Cross-linked pectin could aggregate to form gels and eventually cause phase separation, which was also supported by the syneresis phenomenon observed in the HT-treated puree. After 4 months of refrigerated storage, the viscosity of both HT- and HPP-treated samples did not change significantly; HPP-treated puree still had good liquidity and consistency, while HT-treated puree was a jam-like paste without flow. The high viscosity of HT-treated puree would make it difficult to use in processing, for instance in mixing and piping operations. These results suggest that HPP treatment was able to produce a whole Concord grape puree with a more fresh-like texture and consistency compared to thermally processed grape puree.

### 3.3. Total Phenolic Content (TP), Total Monomeric Anthocyanin Content (TMA), and Antioxidant Activity of Puree Samples

Concord grape and its derivate products are rich in polyphenol compounds, especially flavonoids such as anthocyanins, which contribute to its distinct purple hue. The benefits of Concord grape polyphenol compounds have been reported by many researchers: consumption of Concord grape juice can improve memory in senior adults with cognitive impairment, due to its anti-inflammatory effect and influence on neuronal signaling [10,77]; mice fed with Concord grape juice showed better motor function and cognitive performance related to neuronal and behavioral defects in aging caused by accumulated oxidative stress and inflammation [12]; supplementation of Concord grape juice can help to reduce blood pressure in hypertensive patients [6]; consumption of grapes can reduce the risk of cardiovascular disease [78]; supplementation of 10 $\mathrm{mL}\cdot {\mathrm{kg}}^{-1}\cdot d^{-1}$ Concord grape juice can achieve the same level of antioxidant capacity and protection of low-density lipoprotein against oxidation as that obtained with 400 IU α-tocopherol/d supplementation in healthy adults, and significantly lower native plasma protein oxidation rate than that in α-tocopherol-treated group [79]. Results of total phenolic and total anthocyanin contents and total antioxidant activity of whole Concord puree samples are presented in Table 1.

The TP and TMA values in untreated Concord grape puree were 3.0 ± 0.1 mg/g as gallic acid equivalent (GAE) and 628 ± 35 mg/kg as cyanidin-3-glucoside equivalent (CGE), respectively (Table 1). These results agree with the TP values (2.9 mg/g as GAE) reported for Grenache grape, which is a widely planted red wine grape variety [80]. The TMA value was within the detected range of red grape varieties (40.3 mg/kg to 990.8 mg/kg fresh weight) prepared without seeds [3]. After HPP treatment, the TP value of fresh-made puree was significantly (*p* < 0.05) higher than that of control puree, while there were no significant differences in TMA values among different treatments. Significant increases in phenolic content after HPP treatment were also reported in strawberry puree and blackberry puree [38]. The stated reason for the increased phenolic content in HPP-treated samples is that the high pressure prompted mass transfer, cell membrane permeability, and the release of bound phenolic compounds [81]. After 4 months of refrigerated storage, the TP values in HPP-treated samples significantly decreased from 3.8 mg/g to 2.6 mg/g, while those in HT-treated samples decreased insignificantly from 3.6 mg/g to 2.9 mg/g. The TMA values in both HPP- and HT-treated samples did not show significant changes after 4 months of refrigerated storage. PPO and POD are deleterious enzymes that catalyze the oxidation of phenols, which leads to quality degradation in fruit products, such as discoloration [82]. The greater decrease in phenolic compounds in the HPP samples was attributed to the higher PPO and POD residual enzyme activities (75.2% and 80.7%, respectively) compared to the HT-treated samples (22.6% and 10.2%, respectively). Nevertheless, there were no significant differences in either TP or TMA values between HPP- and HT-treated samples after 4 months of refrigerated storage, probably due to the low reaction rate under refrigerated conditions. These results revealed that HPP is a feasible processing approach to preserve as many nutritive components in Concord grape puree as the conventional thermal processing with extended shelf life, even though HPP was not effective in inactivating deleterious enzymes compared to thermal processing.

Antioxidant activity was determined by the DPPH and ABTS assays and shown as the capacity of scavenging free radicals expressed as Trolox equivalent antioxidant capacity (TEAC). Higher TEAC values indicate higher antioxidant activities. The TEAC results in Table 1 range from 12.2 to 42.1 µmol/g FW, which are higher than the Trolox equivalent (TE) values determined in the supernatant of Concord grape slurry (5 to 20 µmol TE/g FW) [83]. As shown in Table 1, HPP- and HT-treated samples had higher TEAC values than fresh-made control samples, probably due to the release of bound phenolic substances after treatment. No significant differences were found between HPP- and HT-treated samples in antioxidant activity on day 1 and after 4 months of refrigerated storage. After 4 months, the antioxidant capacity in both HPP- and HT-treated samples remained at similar level as in freshly made control puree.

Correlations between antioxidant content (TP and TMA) and antioxidant activity (DPPH and ABTS) were checked using Pearson’s correlation test. The correlations between antioxidant contents and DPPH were not significant (R = 0.185 for TP and R = 0.284 for TMA). The ABTS results showed stronger correlations (R = 0.661 and 0.604, respectively) with both TP and TMA at a significance level of 0.01. Previous studies have reported that antioxidant activity had a strong correlation with phenolic compounds in fruits, grape juice, and red wine [84,85,86,87,88,89]. In our study, the ABTS assay seemed to be a more precise method for determining the antioxidant capacity of the puree product. Floegel et al. [88] also reported the ABTS assay to be a better method compared to DPPH to assess the antioxidant activity of fruits, especially those rich in pigments and hydrophilic antioxidants, after testing 50 antioxidant-rich fruits, vegetables, and beverages. In the evaluation of 16 red grape cultivars, Orak [3] reported that antioxidant activity had significant positive correlations with total phenolic content (R = 0.806) and total anthocyanins (R = 0.455) (*p* = 0.01). However, Kallithraka et al. [89] reported that antioxidant activity had a statistically insignificant correlation with total anthocyanin content after analyzing the grape skin extracts from 17 red grape varieties. In our study, as the Concord grape puree contained the whole grape with skin, pulp, and seeds, it is reasonable that the complex variety and rich amounts of phenolic compounds in the puree matrix led to significant positive correlations not only between antioxidant activity and total phenolics, but also between antioxidant activity and total anthocyanins.

### 3.4. PPO and POD Enzyme Activity

**Figure 4:**
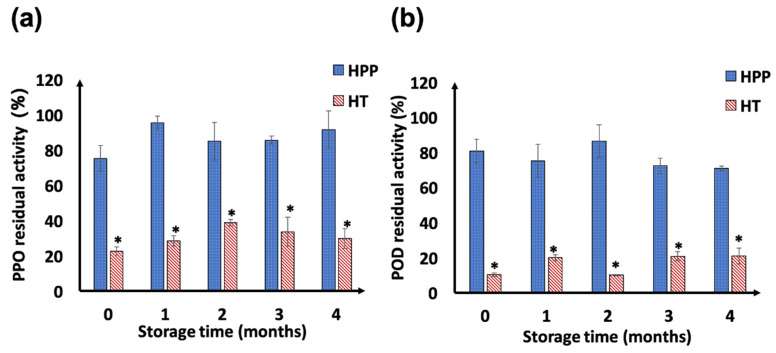
Changes of PPO (**a**) and POD (**b**) residual enzyme activities in HPP- (600 MPa, 3 min) and HT-treated (63 °C, 3 min) purees during 4 months of storage at 4 °C. Denotation of “*” indicates significant difference between different treatments at each sampling point (*p* < 0.05).

The effects of high-pressure and thermal treatment on the PPO and POD enzyme activities of puree are presented in Figure 4a,b, respectively. Compared to the PPO and POD activities in the freshly made control sample, the PPO residual activities in HPP and HT samples were 75.2 ± 7.4% and 22.6 ± 2.4%, while the POD values in HPP and HT samples were 80.7 ± 6.8% and 10.2 ± 1.1%, respectively. During refrigerated storage, both PPO and POD enzyme activity in puree samples did not change significantly. The significantly lower enzyme activity in HT samples indicates that the oxidative enzymes in the grape puree are more sensitive to heat than high pressure. Similar inactivation effects of HPP treatment on oxidative enzymes have been reported in previous studies. Castellari et al. [43] reported that the Trebbiano grape still had 90.7 ± 5.6% PPO activity after HPP (600 MPa, 6 min, 4–22 °C) treatment. Yen and Lin [90] conducted HPP (600 MPa, 25 °C, 15 min) on guava puree and achieved a residual activity of 63% for PPO and 74% for POD; during 60 days of storage at 4 °C, the PPO and POD activities increased gradually and reached 81% and 83%, respectively. Chakraborty et al. [91] reported that 25% inactivation of PPO and POD activity by HPP (500 MPa, 15 min, 30 °C, pH 3.5) can be achieved in treated pineapple puree. Consequently, the total phenolic content and antioxidant activity (by ABTS assay) decreased significantly in HPP samples after a refrigerated storage of 4 months, as shown in Section 3.3. However, the overall quality and antioxidant activity of HPP-treated puree remained comparable to the freshly made samples even after 4 months of storage, suggesting that HPP was able to preserve the grape puree quality despite its inefficiency in enzyme inactivation.

### 3.5. Proximate Composition Analysis

**Figure 5:**
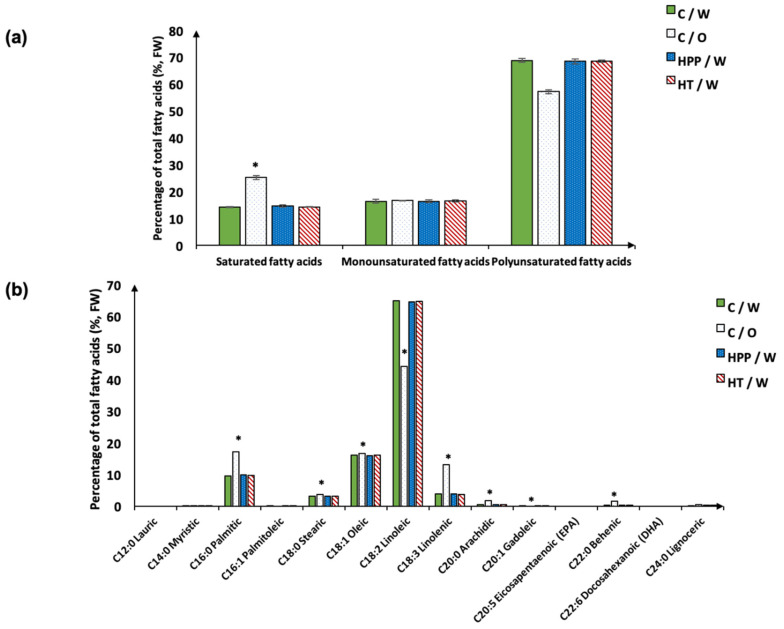
Total fatty acid profile: percentage of saturated fatty acids, monounsaturated fatty acids, and polyunsaturated fatty acids (**a**) and percentage of individual fatty acid (**b**) in Concord grape purees made via different processes. Denotation of “*” indicates significant difference among different treatments (*p* < 0.001). FW: fresh basis; C/O: control puree made without seeds; C/W: control puree made with seeds; HPP/W: HPP(600 MPa, 3 min) puree made with seeds; HT/W: HT (63 °C, 3 min) puree made with seeds.

The aim of this study was to produce a nutritious grape product that utilizes all the potential bioactive components of the Concord grape with minimal processing and no waste. Therefore, an analysis of the composition and nutritional values of the puree product was imperative. Proximate composition and the fatty acid profiles of the untreated control samples with seeds (C/W), untreated samples without seeds (C/O), HPP-treated samples with seeds (HPP/W), and HT-treated samples with seeds (HT/W) are presented in Table 2 and Figure 5, respectively. The moisture and dry matter content in puree made with seeds were around 75% and 24%, respectively. When analyzed on a fresh basis, the moisture content in C/O was significantly higher than in the samples made with seeds, and, accordingly, the dry matter in C/O was significantly lower than in samples made with seeds (*p* < 0.001). Crude protein, crude fiber, total fatty acid, rumen unsaturated fatty acid (RUFAL) and manganese values in puree samples made with seeds were significantly higher than in C/O samples (*p* < 0.001). Crude fiber content in puree samples made with seeds was about 5 times higher than the value in samples made without seeds. In addition to crude fiber, grape pomace also contains antioxidant dietary fiber, such as condensed tannins, which are the main constituent (16% in white grape seeds) of nonextractable polyphenols in grape pomace [92]. These polymetric tannins can lower the cholesterol absorbed by the rats fed with high-cholesterol diets [93], showing potential use in preventing cardiovascular disease. HPP/W and HT/W samples had significantly higher phosphorus contents than the C/O sample (*p* < 0.05); the HPP/W sample had higher copper content than both the C/W and C/O samples (*p* < 0.05). No significant differences in water-soluble carbohydrates (WSC), ash, magnesium, potassium, sodium, iron, zinc, or molybdenum were found among different groups. Grape seeds account for 38–52% of the dry weight of pomace [94]. Grape seeds are mainly (*w*/*w*) composed of 40% fiber, 16% fatty acids, 11% protein, 7% phenolic compounds, and other minor components including sugars and minerals [95]. Grape pomace consists of over 60% (dry matter) indigestible components, including not only dietary fiber, but also condensed tannin and resistant protein, which is unusual compared to other dietary-fiber-rich vegetables; moreover, these unique tannins and protein show distinct physiological and nutritional properties [92]. Therefore, incorporating seeds and skin in the puree product significantly improved the contents of functional components which resulted in a healthier product.

When analyzed as dry matter (Supplementary Table S2), the crude protein and manganese contents in C/O samples were significantly lower than those in the puree samples made with seeds, regardless of the treatment (*p* < 0.05). Calcium, phosphorus, crude fiber, total fatty acids, and RUFAL in puree samples made with seeds were significantly higher than in C/O samples (*p* < 0.001). Insignificant differences of WSC, ash, sodium, iron, zinc, and molybdenum among different groups were found.

As shown in Figure 5, in grape puree samples made with seeds, polyunsaturated fatty acids (PUFAs) accounted for 68% (*w*/*w*) of the total fatty acids, almost 5 times higher than the amount of saturated fatty acids, while this ratio was only about 2 times in seedless puree. The most abundant fatty acid in the Concord grape puree was linoleic acid (LA), which accounted for 65% in the puree made with seeds and 44% in the C/O samples. Lutterodt et al. [96] reported that LA was the major fatty acid, accounting for 75.3% (*w*/*w*), in cold-pressed Concord grape seed oil. PUFAs are essential nutrients and have important effects in the prevention and treatment of coronary heart disease, while LA, also known as omega-6, is the primary and the basis of the *n*-6 fatty acids of PUFAs [97]. C/O samples had significantly lower levels of LA than those made with seeds (*p* < 0.001), suggesting that mixing grape seeds into whole Concord grape puree products provides more dietary and nutritional benefits, especially in providing essential fatty acids, compared to traditional fruit puree products.

### 3.6. Sensory Study

**Figure 6:**
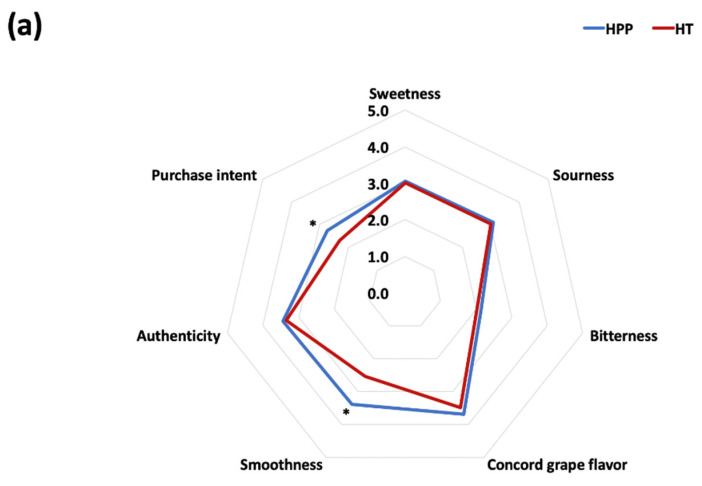

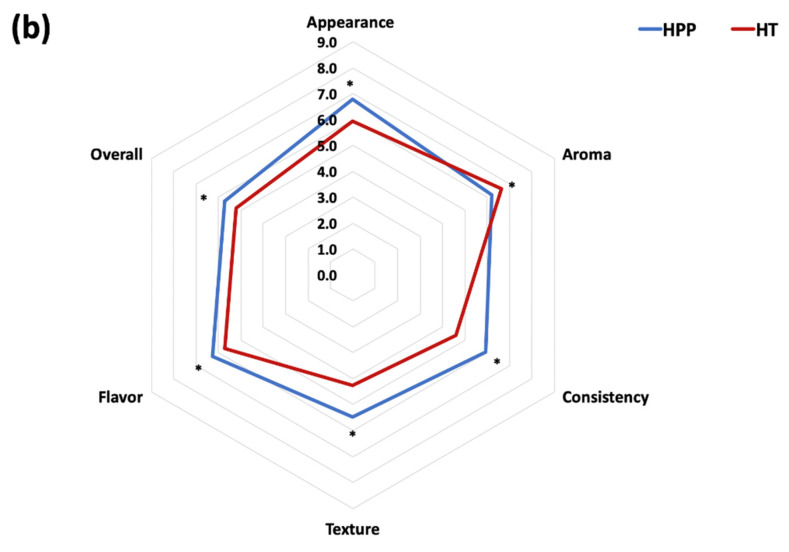
Sensorial attributes of HPP- (600 MPa, 3 min) and HT-treated (63 °C, 3 min) puree: intensity of different characteristics (**a**) on a 5- point hedonic scale (1 = low, 5 = high) and consumer liking (**b**) on a 9-point hedonic scale (1 = dislike it extremely, 9 = like it extremely). Denotation of “*” indicates significant difference between different treatments (*n* = 101, *p* < 0.05).

Sensory evaluation of the puree products was conducted to assess the consumer acceptability of the whole Concord grape puree samples (Figure 6). According to Figure 6b, the HPP-treated sample obtained significantly higher scores than HT-treated samples in all liking categories except for aroma. The higher level of aroma perceived in the HT samples was probably induced by the higher hydrolysis rate of aroma precursors during heating. It was reported in apple juice and nectar that pasteurization (80 °C, 2 min) led to the development of more aromatic compounds [98]. Moreover, consumers’ familiarity with the traditional cooked flavor may have impacted the aroma ratings. After 4 months of storage, however, HPP-treated puree still had the attractive “foxy” (floral and fruity) aroma of Concord grape, while little aroma could be detected in the heated samples, based on informal evaluation. Sweetness, sourness, and flavor are important attributes in evaluating the fruity taste of fruit products. There were no significant differences in any tested intensity categories except for smoothness and purchase intent (Figure 6a). HPP (600 MPa, 3 min) was able to provide a similar taste profile to the heated samples, as there were no significant differences in the sweetness and sourness ratings. Moreover, the low bitterness rating indicated that inclusion of seeds and skin in the product did not compromise the taste profile with skin and seed tannins. The most obvious difference between HPP- and HT-treated samples was based on texture, which affects the consistency, visual color, mouth-feel, and appearance. HPP-treated samples had significantly higher ratings in “smoothness” than HT-treated samples (*p* < 0.05). For the color attribute, color ratings using the color board showed that the HPP sample was perceived as darker (79.2% panelists chose the darkest color on the board) compared to the HT sample (74.3% panelists chose the darkest color on the board). When assessing color preference using the JAR scale, there was no significant difference (for the HT sample the value was 2.5 ± 0.6, while that for the HPP was 2.4 ± 0.7). When comparing the appearance (Figure S2), HPP-treated puree had better consistency and a more homogenous appearance, while the HT-treated sample had an uneven surface (lumpy appearance). These results led to a decline in consumer acceptability for the HT-treated puree, according to the panelists’ comments. HPP samples had significantly higher overall product liking, purchase intent, and product preference ranking (59% of panelists ranked it first) than the HT samples (*p* < 0.05). The HPP treatment delivered a whole Concord grape puree product with better consumer acceptability than the conventional thermal processing, although the sensory results indicate that the puree is more suited as an ingredient (the traditional use) than as a stand-alone product, due to the positive but modest ratings obtained.

## 4. Conclusions

This study compared the quality of HPP- (600 MPa, 3 min, 5 °C) and HT-treated (63 °C, 3 min) whole Concord grape purees during refrigerated storage. HPP was more effective in achieving lower microbial counts, thus providing an extended shelf life of at least 5 months. There were no significant changes in color values, total phenolic, and total anthocyanin contents between HPP and HT samples after 4 months of refrigerated storage. The HPP-treated puree had significantly higher overall liking and purchase intent due to its fresh-like appearance and better consistency compared to the thermally treated puree, while providing a similar taste profile. Proximate composition analysis revealed that incorporating seed and skin into the whole Concord grape puree significantly increased the crude fiber, protein, total fatty acid, and linoleic acid contents while eliminating waste. This study provides a sustainable way to create a bioactive-compound-rich product containing carbohydrates, protein, fiber, and essential fatty acids. However, the textural and flavor changes observed after different processes and during refrigerated storage need to be further investigated. Future studies are needed to better understand the nutritional and sensorial qualities of this novel product, such as the effect of soluble and insoluble tannins on digestibility and gut microbiome and textural changes due to pectin methylesterase activity, as well as changes in aroma compounds.

## Figures and Tables

**Table 1:** Changes in total phenolic (TP) compounds content, total monomeric anthocyanins (TMA) content, and antioxidant activity of whole Concord grape purees during 4 months of storage at 4 °C.

Storage Time (Months)						
		0	1	2	3	4
**TP (mg/g as GAE)**	**Control**	3.0 ± 0.1 a	-	-	-	-
	**HPP**	3.8 ± 0.5 bX	3.0 ± 0.6 aY	2.9 ± 0.2 aY	3.2 ± 0.3 aXY	2.6 ± 0.0 aY
	**HT**	3.6 ± 0.0 abX	3.6 ± 1.0 aX	2.9 ± 0.3 aX	3.2 ± 0.7 aX	2.9 ± 0.3 aX
**TMA (mg/kg as CGE)**	**Control**	628 ± 35 a	-	-	-	-
	**HPP**	620 ± 110 aX	590 ± 40 aX	510 ± 50 aX	520 ± 60 aX	560 ± 30 aX
	**HT**	790 ± 120 aX	840 ± 40 bX	650 ± 170 aX	610 ± 180 aX	610 ± 170 aX
**DPPH (TEAC µmol/g)**	**Control**	12.6 ± 0.3 a	-	-	-	-
	**HPP**	12.7 ± 0.1 aY	13.2 ± 0.1 aX	12.2 ± 0.2 aZ	12.7 ± 0.2 aY	12.6 ± 0.1 aY
	**HT**	13.4 ± 0.1 aX	13.2 ± 0.1 aX	12.8 ± 0.2 aY	12.8 ± 0.1 aY	12.8 ± 0.1 aY
**ABTS (TEAC µmol/g)**	**Control**	34.7 ± 0.6 a	-	-	-	-
	**HPP**	38.1 ± 0.2 bW	36.6 ± 0.5 aX	32.3 ± 0.5 aZ	36.6 ± 0.7 aX	33.9 ± 0.5 aY
	**HT**	37.8 ± 0.5 bY	42.1 ± 0.6 bX	36.1 ± 1.0 bY	36 ± 0.4 aY	33.6 ± 1.4 aZ

Different lowercase letters indicate significant differences among treatments at each predefined sampling point; different uppercase letters indicate significant differences during storage for HPP (600 MPa, 3 min) or HT (63 °C, 3 min) samples (*p* < 0.05). GAE: gallic acid equivalent; CGE: cyanidin-3-glucoside equivalent. TEAC: trolox equivalent antioxidant capacity. “-” means not determined.

**Table 2:** Proximate composition analysis of Concord grape purees made via different processes.

	Fresh Basis			
	Control/W	Control/O	HPP/W	HT/W
**Moisture (%)**	75.90 ± 0.33 a	78.40 ± 0.16 b	75.33 ± 0.12 a	75.07 ± 0.38 a
**Dry matter (%)**	24.10 ± 0.33 a	21.60 ± 0.16 b	24.67 ± 0.12 a	24.93 ± 0.38 a
**Crude protein (%)**	0.93 ± 0.05 a	0.70 ± 0.00 b	0.90 ± 0.00 a	0.97 ± 0.05 a
**Crude Fiber (%)**	2.53 ± 0.05 a	0.53 ± 0.05 b	2.50 ± 0.00 a	2.53 ± 0.09 a
**WSC (%)**	14.43 ± 0.52 A	13.97 ± 0.73 A	14.67 ± 1.05 A	15.80 ± 1.44 A
**Total Fatty acids (%)**	0.29 ± 0.02 a	0.06 ± 0.01 b	0.28 ± 0.02 a	0.32 ± 0.01 a
**RUFAL (%)**	0.24 ± 0.02 a	0.05 ± 0.01 b	0.23 ± 0.02 a	0.27 ± 0.01 a
**Ash (%)**	0.93 ± 0.09 A	1.14 ± 0.22 A	0.90 ± 0.12 A	1.13 ± 0.09 A
**Calcium (%)**	0.02 ± 0.00 A	0.01 ± 0.00 B	0.02 ± 0.00 A	0.02 ± 0.00 A
**Phosphorus (%)**	0.02 ± 0.00 AB	0.02 ± 0.00 A	0.03 ± 0.00 B	0.03 ± 0.00 B
**Magnesium (%)**	0.01 ± 0.00 A	0.01 ± 0.00 A	0.01 ± 0.00 A	0.01 ± 0.00 A
**Potassium (%)**	0.34 ± 0.01 A	0.35 ± 0.00 A	0.32 ± 0.02 A	0.36 ± 0.00 A
**Sodium (%)**	0.00 ± 0.00 A	0.00 ± 0.00 A	0.00 ± 0.00 A	0.00 ± 0.00 A
**Iron (ppm)**	6.33 ± 3.30 A	2.67 ± 0.94 A	5.00 ± 0.82 A	3.67 ± 0.47 A
**Zinc (ppm)**	<1.00 A	<1.00 A	<1.00 A	<1.00 A
**Copper (ppm)**	1.00 ± 0.00 A	1.00 ± 0.00 A	2.00 ± 0.00 B	1.67 ± 0.47 AB
**Manganese (ppm)**	3.00 ± 0.00 A	2.00 ± 0.00 B	3.00 ± 0.00 A	3.00 ± 0.00 A
**Molybdenum (ppm)**	<1.00 A	<1.00 A	<1.00 A	<1.00 A

Different uppercase letters indicate significant differences among different treatments (*p* < 0.05); different lowercase letters indicate significant differences among different treatments (*p* < 0.001). Control/O: untreated samples without seeds; Control/W: untreated samples with seeds; HPP/W: HPP-treated (600 MPa, 3 min) samples with seeds; HT/W: HT-treated (63 °C, 3 min) samples with seeds; WSC: water-soluble carbohydrate; TFA: total fatty acids; RUFAL: rumen unsaturated fatty acids.

## Data Availability

The data presented in this study are available on request from the corresponding author. The data are not publicly available due to the confidential agreement with the sponsor.

## Supplementary Materials

The following are available online at https://www.mdpi.com/article/10.3390/foods10112608/s1, Table S1: Color attributes of control, HPP- and HT- treated puree during 4 months of storage at 4 °C; Figure S1: Results of serum separation rate (SSR, %) of control, HPP- (600 MPa, 3 min) and HT- (63 °C, 3 min) treated sample during 3-month storage at 4 °C; Table S2: Proximate composition analysis of Concord grape puree made by different processes (as dry matter); Figure S2: Particle size distribution (top) and visual appearance (bottom) of control, HPP- (600 MPa, 3 min) and HT- (63 °C, 3 min) treated puree at day 1 (a) and after 4-month refrigerated storage (b).
