# Supplementary material for: High Pressure Processing vs. Thermal Pasteurization of Whole Concord Grape Puree: Effect on Nutritional Value, Quality Parameters and Refrigerated Shelf Life
Source: Foods. 2021 Oct 28;10(11):2608. doi: 10.3390/foods10112608 (PMC8620349; doi:10.3390/foods10112608)
Supplement: Supplementary file 1 [file foods-10-02608-s001.zip › foods-1346685-supplementary.pdf]

Supplementary data

Table S1. Color attributes of control, HPP- and HT- treated puree during 4 months of storage at 4 °C.

Color characteristics		Storage time (months) 0	1	2	3	4
L*	Control	10.9 ± 0.5 a	-	-	-	-
	HPP	10.7 ± 0.1 aXY	10.3 ± 0.1 aZ	10.6 ± 0.1 aYZ	11.1 ± 0.2 aX	10.9 ± 0.3 aXY
	HT	10.1 ± 0.5 aXY	9.1 ± 0.3 bX	10.8 ± 0.3 aY	10.9 ± 0.6 aY	11.3 ± 0.8 aY
a*	Control	16.7 ± 0.4 a	-	-	-	-
	HPP	16.1 ± 0.4 aXY	16.5 ± 0.1 aXY	16.0 ± 0.1 aX	16.7 ± 0.2 aY	16.6 ± 0.2 aY
	HT	17.5 ± 0.1 aX	17.6 ± 0.3 bX	17.1 ± 0.1 bX	17.3 ± 0.4 aX	17.1 ± 0.1 bX
b*	Control	2.9 ± 0.2 ab	-	-	-	-
	HPP	2.7 ± 0.1 aX	2.7 ± 0.1 aX	2.8 ± 0.0 aX	3.3 ± 0.2 aY	3.0 ± 0.2 aXY
	HT	3.5 ± 0.3 bZ	3.5 ± 0.0 bZ	3.7 ± 0.1 bYZ	4.1 ± 0.0 bX	4.0 ± 0.1 bXY
△E	HPP	1.4 ± 0.0 aX	1.8 ± 0.1 aY	1.4 ± 0.1 aXY	1.0 ± 0.3 aX	1.3 ± 0.2 aX
	HT	2.1 ± 0.4 aXY	3.1 ± 0.4 bX	1.5 ± 0.4 aY	1.7 ± 0.6 aY	1.4 ± 0.4 aY
BI	Control	119.3 ± 7.1 a	-	-	-	-
	HPP	115.0 ± 3.4 aX	121.5 ± 0.5 aY	118.0 ± 1.2 aXY	122.3 ± 3.1 aY	120.1 ± 1.0 aXY
	HT	138.0 ± 2.4 bY	153.8 ± 5.9 bX	132.3 ± 6.3 bY	138.8 ± 8.9 bXY	131.7 ± 7.0 bY

[a] Different lowercase letters indicate significant difference among treatments at each predefined sampling point; different uppercase letters indicate significant difference during storage for HPP- (600 MPa, 3 min) or HT- (63 °C, 3 min) treated sample.

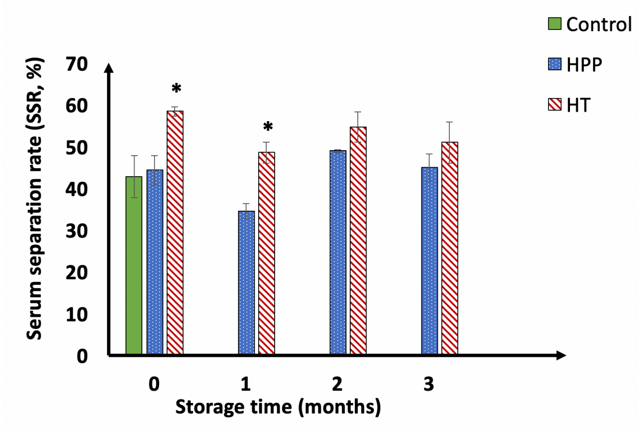

Figure S1. Results of serum separation rate (SSR, %) of control, HPP- (600 MPa, 3 min) and HT- (63 °C, 3 min) treated sample during 3-month storage at 4 °C.

[a] Denotation of "*" indicates significant difference among treatments (p<0.05) at each sampling point.

Table S2. Proximate composition analysis of Concord grape puree made by different processes (as dry matter).

	Dry matter			
	Contrl / W	Control /O	HPP / W	HT / W
**Moisture (%)**	-	-	-	-
**Dry matter (%)**	-	-	-	-
**Crude protein (%)**	3.90 ± 0.16 A	3.23 ± 0.05 B	3.67 ± 0.05 A	3.90 ± 0.14 A
**Crude Fiber (%)**	10.63 ± 0.33 a	2.50 ± 0.22 b	10.07 ± 0.05 a	10.17 ± 0.48 a
**WSC (%)**	59.93 ± 3.03 A	64.77 ± 3.89 A	59.37 ± 4.11 A	63.40 ± 5.51 A
**Total Fatty acids (%)**	1.19 ± 0.13 a	0.29 ± 0.05 b	1.12 ± 0.09 a	1.26 ± 0.05 a
**RUFAL (%)**	1.01 ± 0.11 a	0.21 ± 0.04 b	0.95 ± 0.07 a	1.07 ± 0.04 a
**Ash (%)**	3.87 ± 0.40 A	5.27 ± 1.01 A	3.67 ± 0.47 A	4.51 ± 0.31 A
**Calcium (%)**	0.07 ± 0.00 a	0.05 ± 0.00 b	0.08 ± 0.00 a	0.08 ± 0.00 a
**Phosphorus (%)**	0.10 ± 0.00 a	0.08 ± 0.00 b	0.10 ± 0.00 a	0.10 ± 0.00 a
**Magnesium (%)**	0.05 ± 0.00 A	0.04 ± 0.00 B	0.05 ± 0.00 A	0.05 ± 0.00 A
**Potassium (%)**	1.39 ± 0.04 A	1.63 ± 0.00 B	1.3 ± 0.08 A	1.45 ± 0.03 A
**Sodium (%)**	0.00 ± 0.00 A	0.00 ± 0.00 A	0.01 ± 0.00 A	0.00 ± 0.00 A
**Iron (ppm)**	25.33 ± 13.22 A	13.00 ± 2.83 A	20.67 ± 3.40 A	13.67 ± 1.25 A
**Zinc (ppm)**	4.00 ± 0.00 A	3.67 ± 0.47 A	3.33 ± 0.47 A	4.00 ± 0.82 A
**Copper (ppm)**	6.00 ± 0.00 A	6.00 ± 0.00 A	7.00 ± 0.00 B	6.00 ± 0.00 A
**Manganese (ppm)**	11.00 ± 0.00 A	8.67 ± 0.47 B	10.67 ± 0.47 A	11.33 ± 0.47 A
**Molybdenum (ppm)**	<1.00 A	<1.00 A	<1.00 A	<1.00 A

[a]Different uppercase letters indicate significant differences among different treatments ($p<0.05$); different lowercase letters indicate significant differences among different treatments ($p<0.001$). Control/O: untreated samples without seeds; Control/W: untreated samples with seeds; HPP/W: HPP- (600 MPa, 3 min) treated samples with seeds; HT/W: HT- (63 °C, 3 min) treated samples with seeds; WSC: water soluble carbohydrate; TFA: total fatty acids; RUFAL: rumen unsaturated fatty acids. "-" means not determined.

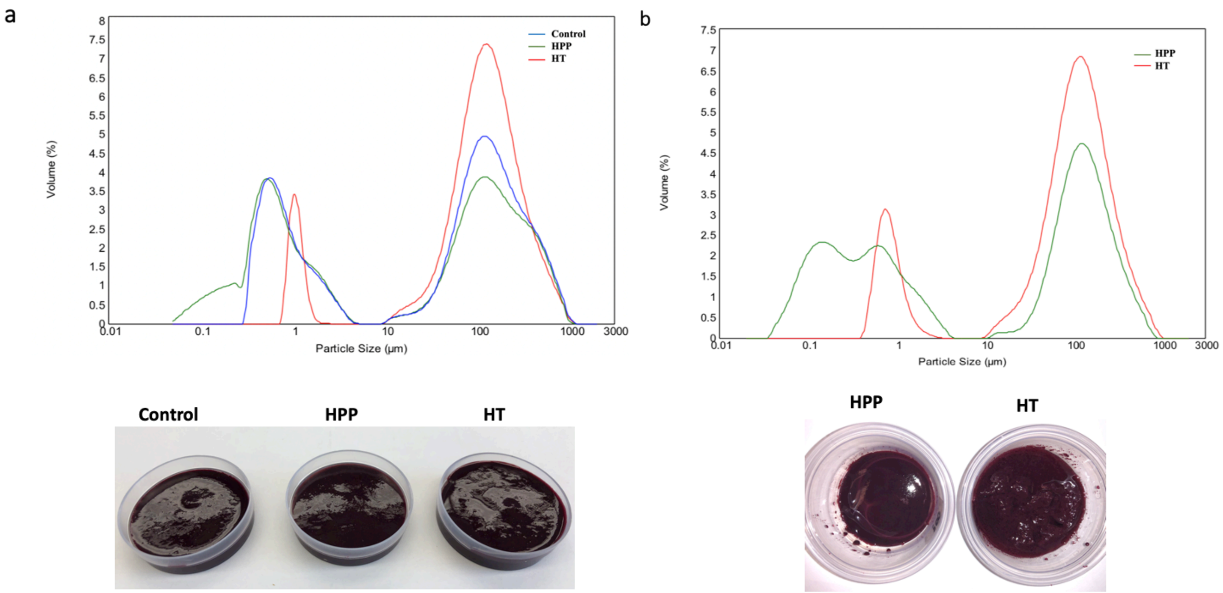

Figure S2. Particle size distribution (top) and visual appearance (bottom) of control, HPP- (600 MPa, 3 min) and HT- (63 °C, 3 min) treated puree at day 1 (a) and after 4-month refrigerated storage (b).
